# Multimodal [^18^F]FDG PET/CT Is a Direct Readout for Inflammatory Bone Repair: A Longitudinal Study in TNFα Transgenic Mice

**DOI:** 10.1002/jbmr.3748

**Published:** 2019-07-30

**Authors:** Silvia Hayer, Markus Zeilinger, Volker Weiss, Monika Dumanic, Markus Seibt, Birgit Niederreiter, Tetyana Shvets, Florian Pichler, Wolfgang Wadsak, Bruno K Podesser, Thomas H Helbich, Marcus Hacker, Josef S Smolen, Kurt Redlich, Markus Mitterhauser

**Affiliations:** ^1^ Division of Rheumatology, Department of Medicine III, Medical University of Vienna Vienna Austria; ^2^ Department of Biomedical Imaging and Image‐guided Therapy Division of Nuclear Medicine, Medical University of Vienna Vienna Austria; ^3^ Faculty of Engineering, University of Applied Sciences Wiener Neustadt Wiener Neustadt Austria; ^4^ Faculty of Health Sciences, University of Applied Sciences Wiener Neustadt Wiener Neustadt Austria; ^5^ Center for Biomarker Research in Medicine (CBmed) Graz Austria; ^6^ Center of Biomedical Research, Medical University of Vienna Vienna Austria; ^7^ Department of Biomedical Imaging and Image‐guided Therapy Division of Molecular and Gender Imaging, Medical University of Vienna Vienna Austria; ^8^ Ludwig Boltzmann Institute of Applied Diagnostics Vienna Austria

**Keywords:** µPET/CT, ^18^F‐FDG, ^18^F‐NaF, experimental arthritis, TNF blockade, bone healing

## Abstract

In rheumatoid arthritis (RA), chronic joint inflammation leading to bone and cartilage damage is the major cause of functional impairment. Whereas reduction of synovitis and blockade of joint damage can be successfully achieved by disease modifying antirheumatic therapies, bone *repair* upon therapeutic interventions has only been rarely reported. The aim of this study was to use fluorodeoxyglucose ([^18^F]FDG) and [^18^F]fluoride µPET/CT imaging to monitor systemic inflammatory and destructive bone remodeling processes as well as potential bone repair in an established mouse model of chronic inflammatory, erosive polyarthritis. Therefore, human tumor necrosis factor transgenic (hTNFtg) mice were treated with infliximab, an anti‐TNF antibody, for 4 weeks. Before and after treatment period, mice received either [^18^F]FDG, for detecting inflammatory processes, or [^18^F]fluoride, for monitoring bone remodeling processes, for PET scans followed by CT scans. Standardized uptake values (SUV_mean_) were analyzed in various joints and histopathological signs of arthritis, joint damage, and repair were assessed. Longitudinal PET/CT scans revealed a significant decrease in [^18^F]FDG SUVs in affected joints demonstrating complete remission of inflammatory processes due to TNF blockade. In contrast, [^18^F]fluoride SUVs could not discriminate between different severities of bone damage in hTNFtg mice. Repeated in vivo CT images proved a structural reversal of preexisting bone erosions after anti‐TNF therapy. Accordingly, histological analysis showed complete resolution of synovial inflammation and healing of bone at sites of former bone erosion. We conclude that in vivo multimodal [^18^F]FDG µPET/CT imaging allows to quantify and monitor inflammation‐mediated bone damage and reveals not only reversal of synovitis but also bone repair upon TNF blockade in experimental arthritis. © 2019 The Authors. *Journal of Bone and Mineral Research* Published by Wiley Periodicals, Inc.

## Introduction

Rheumatoid arthritis (RA) is the most common chronic inflammatory and destructive joint disease, affecting around 0.5% to 1% of the population worldwide.^1^ Perpetuation of inflammatory processes within the synovial tissue leads to local activation of tissue‐degrading enzymes and formation of bone‐resorbing osteoclasts, provoking progressive cartilage and bone destruction.^2^ Therefore, early interference with inflammatory processes and prevention of bone and cartilage destruction are crucial to preserve function in RA. Several studies in both human RA and animal models have shown that therapeutic interventions with biological agents, such as tumor necrosis factor (TNF) or interleukin‐6 receptor (IL‐6R) blockers, lead to the resolution of synovitis as well as inhibition of joint damage progression. However, initiation of bone regeneration processes and repair of joint damage have rarely been reported and need further elucidation.^3^, ^4^, ^5^, ^6^, ^7^

Conventional radiography remains the first choice for the assessment of structural bone and cartilage damage in RA patients. On the other hand, novel imaging methods, such as combined positron emission tomography/computed tomography (PET/CT) provide insights into pathophysiological processes together with anatomical localization. Fluorodeoxyglucose ([^18^F]FDG, 2‐deoxy‐2‐[^18^F]fluoro‐D‐glucose) demonstrated great value for localizing articular inflammatory processes in patients suffering from RA; not only increased uptake of [^18^F]FDG in inflamed joints but also a strong correlation with disease activity was observed.^8^, ^9^, ^10^, ^11^ PET imaging with the bone tracer [^18^F]fluoride showed high bone turnover in diseases like RA, osteoarthritis (OA), and osteoporosis.^12^, ^13^

In experimental models, it has been shown that TNF inhibition can not only prevent progressive bone destruction but also initiate bony healing processes in TNF‐driven arthritis.^14^, ^15^ However, these animal studies were based on histological comparisons after different time points, lacking continuous follow‐up in the same animal. This limitation can be overcome by in vivo imaging techniques using [^18^F]FDG or [^18^F]fluoride.^16^, ^17^, ^18^ However, they have not yet been used for monitoring therapeutic responses in *individual* animals.^19^, ^20^

The aims of our study were (i) the longitudinal characterization of pattern and intensity of joint inflammation in a TNF‐driven erosive mouse model of arthritis; (ii) to differentiate joint inflammation in vivo and bone remodeling processes in vivo from bone damage; and (iii) to evaluate the reversibility of these events in individual animals during therapeutic intervention, such as anti‐TNF antibody (anti‐TNF), using [^18^F]FDG PET, [^18^F]fluoride PET, and CT.

## Materials and Methods

### Animals

Human tumor necrosis factor‐α transgenic mice (hTNFtg; Tg197 strain, C57BL/6 genetic background, originally generated by George Kollias, Fleming Institute, Athens, Greece).^21^ hTNFtg mice develop a chronic inflammatory, erosive, symmetric polyarthritis starting around 4 to 5 weeks after birth.^22^ Mice were maintained under standardized, conventional housing conditions (humidity 50%, 22°C: 12:12 light‐dark cycle). Heterozygous litters were genotyped by PCR of DNA isolated from tail biopsies as described.^21^ Age‐matched non‐transgenic wild type (wt) littermates were used as controls. The local ethical committee from the Austrian Federal Ministry of Education, Science and Research approved all experiments (BMWFW‐66.009/0128/II/3b/2014).

### Clinical assessment of arthritis

Clinical signs of arthritis including paw swelling and grip strength were assessed weekly in front and hind paws in a blinded manner by an independent investigator not involved in treatment using an established semiquantitative scoring system: paw swelling 0 to 3 (0 = no swelling, 3 = severe swelling); grip strength 0 to –3 (0 = no loss of grip strength, –3 = severe loss of grip strength) as described.^14^

### Therapeutic interventions

Therapeutic TNF blockade was performed in 8‐week‐old hTNFtg mice by intraperitoneal administration of anti‐TNF antibody (Remicade [infliximab], 10 mg/kg, Jannsen Biologics B.V., Leiden, Netherlands) three times per week for 4 weeks. Placebo‐treated, sex‐ and age‐matched hTNFtg littermates and wt mice served as controls. Two animal cohorts (*n* = 6 animals per group) were evaluated: cohort 1 received [^18^F]FDG PET/CT scans; cohort 2 received [^18^F]fluoride PET/CT scans. Female mice were used for cohort 1, male littermates for cohort 2. After final PET/CT, animals were anesthetized to collect blood samples, euthanized by cervical dislocation, and joints were isolated for histological and µCT analysis.

### Radiosynthesis

[^18^F]FDG and [^18^F]fluoride were synthesized in house (GE FASTlab®; GE Healthcare, Piscataway, NJ, USA) with dedicated software and single‐use cassettes (FastLab Casettes, GE Healthcare).

### In Vivo µPET/CT

Longitudinal µPET/CT scans were performed before (1 day before treatment) and after the treatment period (day 28). For µPET/CT scans, mice received either [^18^F]FDG (16.96 ± 2.32 MBq; 107 ± 10 µL) or [^18^F]fluoride (18.42 ± 3.04 MBq; 104 ± 5 µL) via retro‐orbital injection under isoflurane‐anesthesia (Inveon microSPECT/PET/CT; Siemens Medical Solutions, Knoxville, TN, USA). A 20‐min static PET scan was performed 50 min postinjection, followed by two consecutive in vivo CT scans. CT acquisition parameters were as follows: 360 projections (full rotation, 360 degrees, 1‐degree projections); settle time 300 ms (hind limbs) 2 × 2 binning (whole body), no binning (hind limbs); 1.5‐mm aluminum filter; exposure time 950 ms; voltage 80 kV; current 500 µA; axial field of view (FOV) 10 cm (whole‐body) and 4 cm (hind limbs). For the evaluation of the pharmacokinetics and the determination of the appropriate tracer equilibrium, a 60‐min dynamic PET acquisition was performed as pilot for both [^18^F]FDG and [^18^F]fluoride. CT data were reconstructed with the Feldkamp algorithm, ramp filter, standard mouse beam‐hardening correction, and noise reduction (matrix size 1024 × 1024; effective pixel size 9.75 µm). Framing: 1 × 30 s, 4 × 60 s, 1 × 90 s, 4 × 120 s, 1 × 210 s, 4 × 300 s, 1 × 450 s, and 4 × 600 s. PET images were reconstructed using an OSEM 3D/OP‐MAP (three‐dimensional Ordered Subset Expectation Maximum with Ordinary Poisson‐Maximum a Posteriori) scatter‐corrected reconstruction algorithm and a ramp filter (matrix size 128 × 128). Data were normalized and corrected for random, dead time, radioactive decay, and weight of the animal. PET data are expressed as mean standardized uptake values (SUV; g/mL). CT data were used for attenuation correction of PET images. Multimodal rigid‐body image registration and biomedical image quantification were performed using Inveon Research Workplace (IRW) and PMOD 3.8 (PMOD Technologies Ltd, Zurich, Switzerland). 3D volumes of interest were drawn using a 3D threshold‐based auto‐segmentation algorithm. Representative 3D images of joints and quantitative changes in bone volume were determined in selected bone regions (patella, femoral epiphysis, talus) using the Multimodal 3D Visualization tool from the IRW Software. Briefly, after manually segmentation of selected bone ROIs and constant setting of threshold levels for bone tissue (higher than 1800 HU) statistics of bone volume (mm^3^) were calculated. Data were obtained from both left and right limbs.

### Ex Vivo µCT

Isolated joints were fixed in 7% formaldehyde overnight and stored in 70% ethanol. µCT scans were performed using a µCT35 (Scanco Medical AG, Brüttisellen, Switzerland) with the following settings: 55 kVp; 145 µA, 8 W; 300 ms; high resolution: trabecular bone microarchitecture, cortical bone density; or medium resolution: talus, ankle, knee, shoulder joints; reconstruction threshold: 300 (talus, ankle), 270 (knee, shoulder); as described.^14^, ^23^

### Histology

Paraffin‐embedded joint sections were stained with hematoxylin and eosin (H&E), toluidine blue (TB), and tartrate‐resistant acid phosphatase (TRAP). TRAP staining identifying bone‐resorbing osteoclasts was performed using a TRAP staining kit (Sigma Diagnostics, Livonia, MI, USA; cat.no.387‐A) according to the manufacturer's procedure. Briefly, deparaffinized sections were incubated with staining solution I (containing naphthol AS‐BI phosphoric acid solution, acetate solution, and tartrate solution) for 1 hour at 37°C in a water bath protected from light. TRAP staining was developed with substrate solution II (fast garnet GBC base solution, sodium nitrite solution) for 5 to 8 min at 37°C and nuclei were counterstained with Meyer's hematoxylin. The number of TRAP+ multinucleated osteoclasts (more than three nuclei) and the area of TB‐stained cartilaginous regeneration tissue (mm^2^) was quantitatively assessed determined using the Osteomeasure software (OsteoMetrics, Decatur, GA, USA). Semiquantitative analysis of histopathologies was assessed as follows: *synovial inflammation*: 0 = none; 1 = mild infiltration of inflammatory cells, thickening of synovial membrane (2 to 3 cell layers); 2 = increased thickening, multilayered synovial cell membrane; 3 = massive accumulation of inflammatory cells throughout entire joints, expanded hyperplasia. *Subchondral bone erosion*: 0 = intact; 1 = small superficial lesions of cortical and subchondral bone; 2 = enhanced focal, subchondral bone erosions, partial or complete penetration of cortical bone; 3 = severe subchondral bone erosions, complete breakthrough to the bone marrow cavity. *Proteoglycan loss* of superficial cartilage layer (blue stained): 0 = intact; 1 = destaining < 25%; 2 = < 50%; 3 = >50% or complete TB destaining. *Degradation of cartilage*: 0 = intact; 1 = < 25% loss of calcified cartilage; 2 = 25% to 50% loss of calcified and/or superficial cartilage; 3 = >50% loss of both layers. Methacrylate‐embedded sections were stained with Goldner and Movat to evaluate osteoid formation and osteoblasts as well as for Kossa to visualize mineralized bone tissue. Briefly, modified MOVAT pentachrome staining was obtained by a mix of five stains including Alcian blue, Weigert hematoxylin, brilliant crocein 0.1% combined with acidic fuchsine (0.1%) in a ratio of eight to two and finally saffron du Gatinois. Kossa staining included silver nitrate (5%), sodium‐formol solution, sodium thiosulfate (5%), and counterstaining with ponceau de xylidine and orange G. Histomorphometric analyses were assessed using the Osteomeasure software as described.^14^, ^24^ Immunohistochemical stainings were performed to identify neutrophil granulocytes (clone 7/4; AbD Serotec, Hercules, CA, USA), macrophages (F4/80ab, clone CI:A3‐1; Serotec), T cells (CD3ab, clone CD3‐12; Serotec), and B cells (CD45R, clone RA3‐6B2; BD Pharmingen, Franklin Lakes, NJ, USA). Relative numbers of cell populations were quantified using TissueQuest software (TissueGnostics GmbH, Vienna, Austria). Immunohistochemical staining for Collagen type II expression was performed after enzymatic antigen retrieval (proteinase type XIV, 1:2000) using collagen type II antibody (Abcam, Cambridge, MA, USA; ab34712), and goat anti‐rabbit IgG (biotinylated, BA‐1000; Vector Laboratories, Burlingame, CA, USA).

Human joint tissue was obtained from RA patients undergoing joint replacement. Patients signed an informed consent prior to the donation of synovial tissues. The study was approved by the ethical committee of the Medical University of Vienna. MOVAT‐stained or TRAP‐stained sections were investigated.

### ELISA

Blood samples were taken to determine expression levels of matrix‐degrading MMP‐3 (1:20 serum dilution; R&D Systems, Minneapolis, MN, USA) according to the manufacturer's protocol.

### Real‐time quantitative polymerase chain reaction

Total RNA was isolated from the front paws. Briefly, after removal from skin and toes carpal tissue was shock‐frozen and stored at –80°C. Frozen tissue was mechanically homogenized using steel beads and a TissueLyser II (Qiagen, Hilden, Germany) in the presence of Trizol reagent (Invitrogen, Carlsbad, CA, USA). Total RNA was extracted according to the Trizol manufacturer's protocol and a subsequent purification step using RNeasy kit (Qiagen). Real‐time quantitative polymerase chain reaction (RT‐qPCR) analyses are described further in the Supporting Methods.

### Statistical analysis

Statistical analyses were done using GraphPad Prism 5 software (GraphPad Software, Inc., La Jolla, CA, USA). Data are expressed as mean ± standard error of the mean (SE). Student's *t* test (two‐tailed) was used to compare individual parameter between two animal groups (unpaired) or to compare longitudinal changes within one group (repeated measurements, paired *t* test). Statistical significance between more than two groups was evaluated with one‐way ANOVA, Tukey post‐test. Linear regression analysis was used to investigate the relationship between two numeric parameters (Pearson correlation). Values of *p* < 0.05 were considered statistically significant (*p < 0.05, **p < 0.005, ***p < 0.001, ****p < 0.0001).

## Results

### [^18^F]FDG PET/CT imaging reveals remission of joint inflammation after TNF blockade

**Figure 1:**
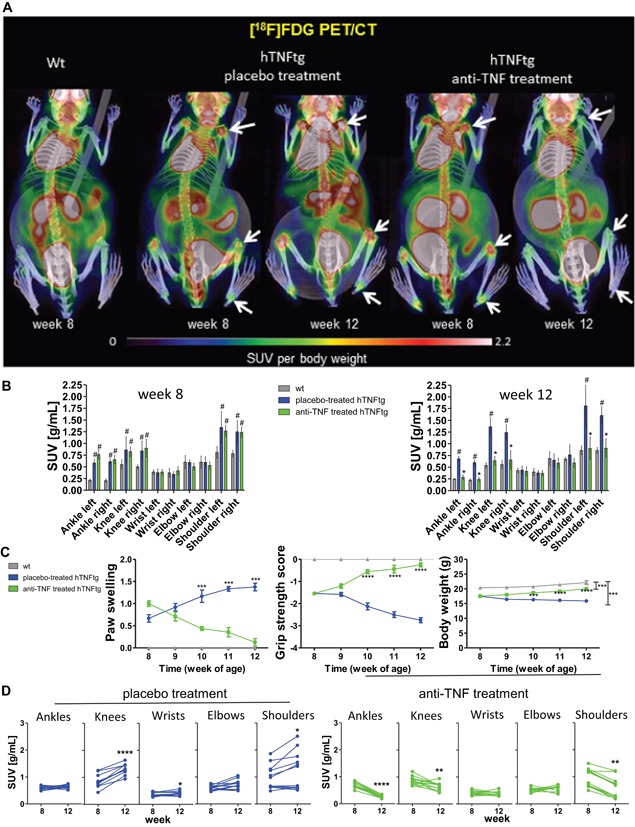
Longitudinal [^18^F]FDG PET/CT monitoring of joint inflammation in anti‐TNF–treated and placebo‐treated hTNFtg animals. (*A*) [^18^F]FDG accumulations in joints (arrows) before and after the treatment period in placebo‐treated hTNFtg, anti‐TNF–treated hTNFtg, and wt mice. (*B*) [^18^F]FDG SUV_mean_ values before and after the treatment of the 3 groups (#statistical significant compared to wt; *statistical significant difference between anti‐TNF and placebo‐treated). (*C*) Clinical course of arthritis signs and body weight during treatment. (*D*) Changes of individual [^18^F]FDG SUV_mean_ values after anti‐TNF or placebo treatment. Data analyzed for clinical course, PET/CT and SUV included 6 placebo‐treated hTNFtg, 6 anti‐TNF–treated hTNFtg, and 4 wt animals. [Color figure can be viewed at wileyonlinelibrary.com]

To assess potential reversibility of disease activity, treatment started at a progressed stage of disease with established joint inflammation and damage. Knowing the precise course of the disease,^22^ we started the treatment in 8‐week‐old hTNFtg mice. At this age, hTNFtg mice exhibited an increased articular [^18^F]FDG accumulation in various joints compared to wt animals (Fig. 1 *A*). Quantitative analysis of [^18^F]FDG SUVs revealed significantly increased values in ankle (3.1‐fold), knee (1.6‐fold), and shoulder (1.6‐fold) in hTNFtg animals compared to wt mice (Fig. 1 *B*, left panel). Small inflammatory areas in wrist or elbow were not resolvable by PET because of partial volume effects (Supporting Fig. 3). At this time point, hTNFtg mice also showed clinical signs of arthritis such as reduced grip strength and increased paw swelling, most prominently in hind paws (Fig. 1 *C*). In histological sections of euthanized littermates, we found synovial inflammation, invasive pannus formation, bone erosions, and cartilage proteoglycan loss (Supporting Fig. 1).

hTNFtg mice then received placebo or anti‐TNF, respectively, for an additional 4 weeks. Placebo‐treated hTNFtg animals exhibited a further increase of joint inflammation as observed by both imaging and clinical assessment (Fig. 1 *A*, *C*). [^18^F]FDG uptake increased 1.5‐fold in knees (*p* < 0.0001) and 1.3‐fold in shoulders (*p* = 0.03) during that period (Fig. 1 *B* [right panel], *D*). Additionally, in wrists, a slight increase of [^18^F]FDG uptake became apparent (1.14‐fold, *p* = 0.03). In contrast, when quantifying the changes of [^18^F]FDG SUVs in hTNFtg animals after anti‐TNF treatment, we observed a significant reduction compared to baseline values in ankles (*p* < 0.0001), knees (*p* = 0.0028), and shoulders (*p* < 0.0001). The reduction of [^18^F]FDG SUVs resulted in values comparable with SUVs in wt animals (Fig. 1 *B*; Supporting Fig. 2), indicating complete remission of joint inflammation (Fig. 1 *D*). Consistently, anti‐TNF therapy revealed clinical improvement with a significant decrease in paw swelling and increase in grip strength (Fig. 1 *C*).

**Figure 2:**
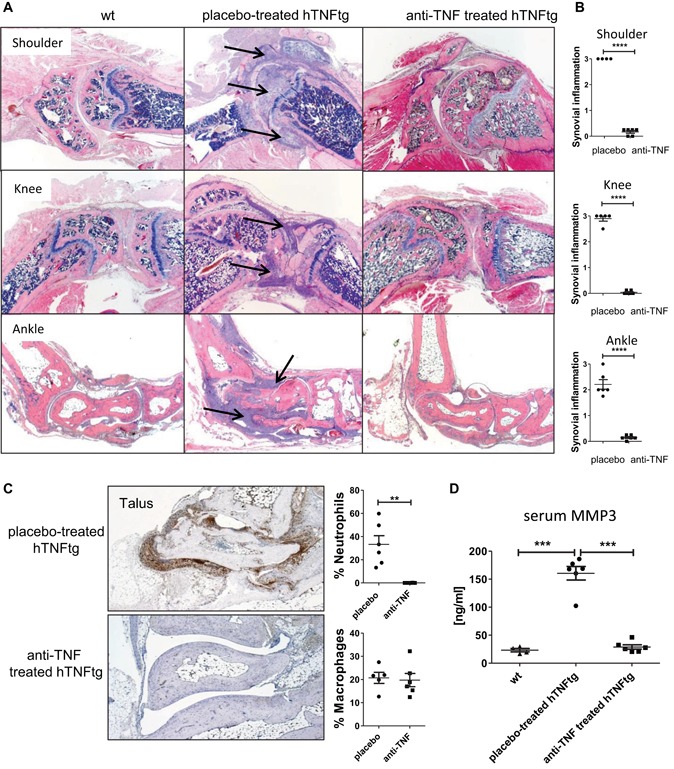
Resolution of synovial inflammation upon anti‐TNF treatment. (*A*) Representative images from synovial inflammation (arrows) in shoulders, knees, and ankles from placebo‐treated and anti‐TNF–treated mice (H&E staining). (*B*) Semiquantitative analysis of synovial inflammation. (*C*) Immunohistochemical staining of neutrophils (brown colored cells) within synovial tissue. Quantitative analysis of relative numbers of neutrophils and macrophages (% of total synovial cells). (*D*) Serum levels of MMP3 (ng/mL). Magnification: (*A*) 25×, (*C*) 50×. Analysis included following number of animals: placebo‐treated hTNFtg, *n* = 6; anti‐TNF–treated, *n* = 6. [Color figure can be viewed at wileyonlinelibrary.com]

To correlate [^18^F]FDG uptake with synovitis, we investigated histological sections from joints after treatment. Placebo‐treated hTNFtg animals showed massive inflammation of the synovial membrane in various joints such as ankles, knees, and shoulders. Synovitis was characterized by synovial hyperplasia, infiltration of inflammatory cells such as macrophages and neutrophils, as well as pannus formation (Fig. 2). TNF blockade led to a complete resolution of these inflammatory processes. Compared to placebo‐treated animals, anti‐TNF–treated hTNFtg mice showed a significant decrease in synovial inflammation (*p* < 0.0001) and infiltrating neutrophil granulocytes (*p* = 0.012) (Fig. 2 *A–C*). The only areas of residual abnormalities were found as slightly thickened, multilayered regions within the synovial membrane without inflammatory cell infiltrates. Serological analyses revealed a significant decrease of matrix‐metalloproteinase (MMP)‐3 levels in anti‐TNF–treated mice compared to placebo‐treated animals (*p* < 0.0001, Fig. 2 *D*).

### CT imaging proves healing of local bone erosions after TNF blockade

**Figure 3:**
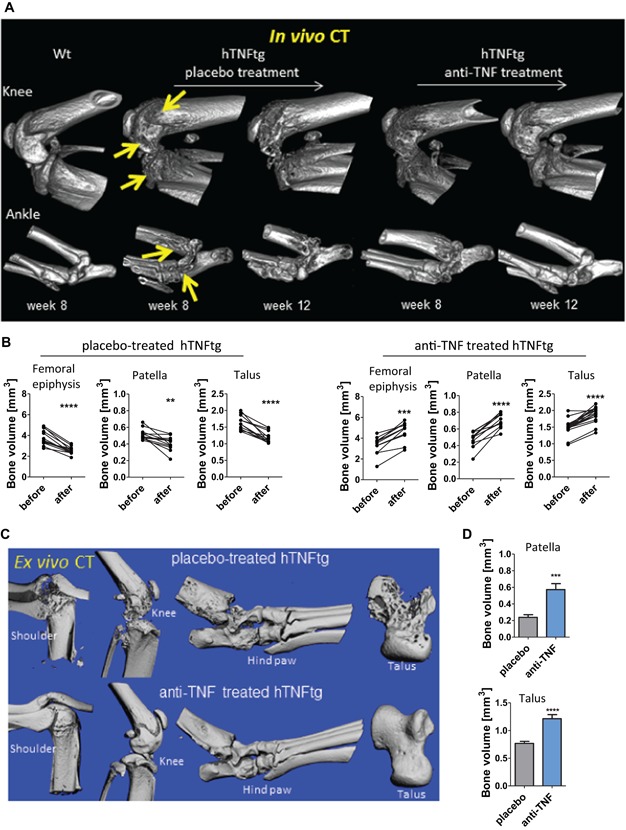
Longitudinal in vivo CT analysis indicates bone repair upon anti‐TNF treatment. (*A*) Repeated in vivo 3D CT images from knee and ankle joints after 8 and 12 weeks: progressive bone destruction in placebo‐treated mice (arrows) and bone repair in anti‐TNF–treated mice. (*B*) Quantitative assessment of bone volume (mm^3^) from distal epiphysis of femur, patella, and talus before and after treatment (evaluated in both right and left limbs). (*C*) Ex vivo µCT images of joints. (*D*) Quantitative µCT analysis of bone mass in right talus and patella indicated by bone volume (in mm^3^). Data were obtained from placebo‐treated hTNFtg (*n* = 6) and anti‐TNF–treated hTNFtg (*n* = 6). [Color figure can be viewed at wileyonlinelibrary.com]

To identify structural bone damage and repair of bone erosions, we assessed in vivo CT scans from hind limbs of placebo‐treated hTNFtg mice, anti‐TNF–treated hTNFtg mice, and wt controls (Supporting Fig. 2). At week 8, hTNFtg mice showed porous, rough, eroded bone surfaces at the sites of ankle and tarsal bones as well as femoral and tibial heads, indicating inflammation‐mediated subchondral and cortical bone erosions (Fig. 3 *A*). At the end of the observation period, damage indicated as loss of bone volume was significantly pronounced in placebo‐treated hTNFtg mice (Fig. 3 *A*, *B*; two‐tailed, paired *t* test, ***p < 0.0001). In contrast, treatment with anti‐TNF led to improved or intact bone architecture and increased bone density with regular, smoother bone surfaces in knee and ankle (Fig. 3A). By volumetric quantification of in vivo CT scans, we found a significant improvement of bone volume in knees and ankles (Fig. 3 *B*; ****p < 0.0001).

Consistent with these in vivo CT data, 3D reconstructed ex vivo µCT images confirmed intact, smooth bone surfaces and structures in joints from anti‐TNF–treated hTNFtg mice. Placebo‐treated hTNFtg animals showed massively destroyed and deformed joints (Fig. 3 *C*). Quantification of the bone volume of the talus and the patella demonstrated a significantly higher bone volume in anti‐TNF–treated compared to placebo‐treated mice (*p* < 0.0001, Fig. 3 *D*).

### TNF blockade fosters formation of fibrocartilaginous refilling and regeneration tissue at bone lesions

**Figure 4:**
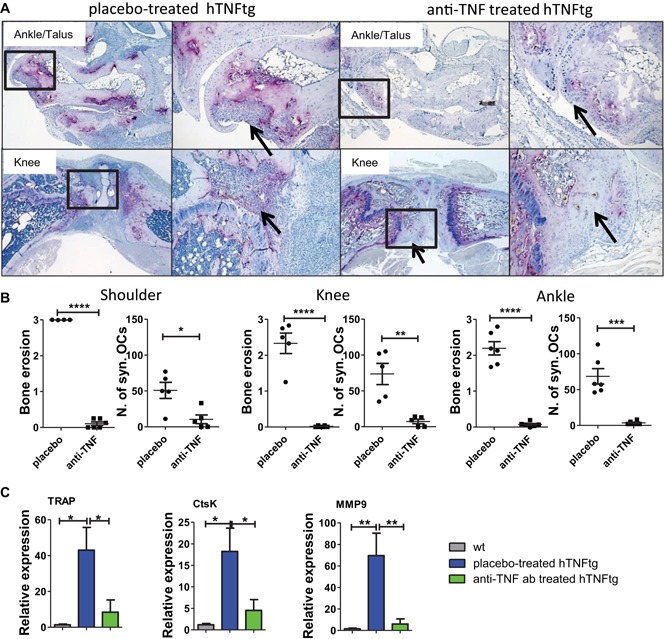
(*A*) Representative images from synovial osteoclasts (TRAP+ purple‐colored cells, >3 nuclei) and subchondral bone erosions (arrow) invaded by synovial pannus in placebo‐treated mice. TNF blockade inhibited osteoclast formation and initiated bone repair (arrows). (*B*) Semiquantitative analysis of subchondral bone erosions and number of synovial osteoclasts. (*C*) mRNA expression levels of osteoclast specific genes such as TRAP, CtsK, and MMP‐9 in paw extracts. Magnification: talus: 50×, 100×; knee: 25×, 100×. Number of animals: placebo‐treated hTNFtg, *n* = 6; anti‐TNF–treated hTNFtg, *n* = 6; wt *n* = 4. CtsK = cathepsin K; MMP‐9 = matrix metalloproteinase 9. [Color figure can be viewed at wileyonlinelibrary.com]

**Figure 5:**
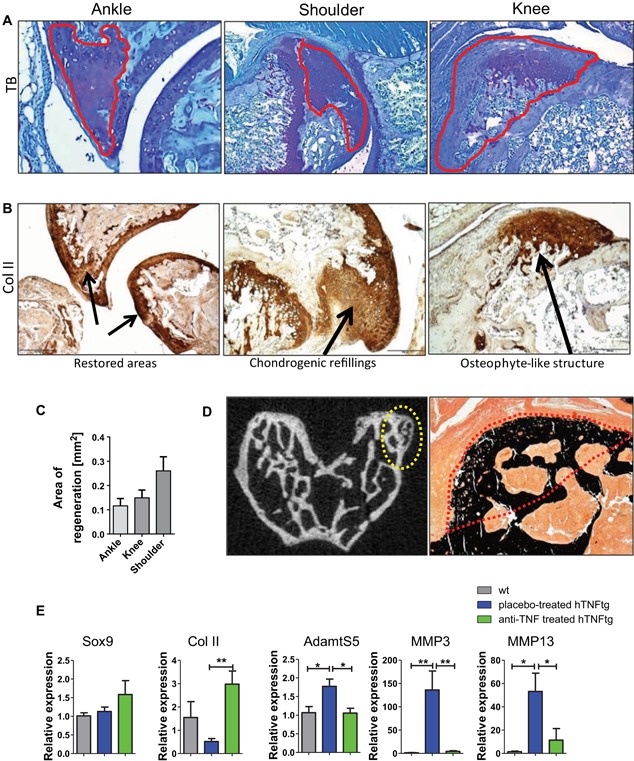
Repair of preexisting bone erosions upon TNF blockade. (*A*) Ongoing healing processes represented by proteoglycan‐rich cartilaginous fillings in TB‐stained sections from anti‐TNF–treated hTNFtg mice. (*B*) Collagen type II (Col II) staining (brown color) indicates restored joint structure, ongoing chondrogenic refillings and osteophyte‐like structures. (*C*) Quantitative assessment of TB stained regeneration area (mm^2^). (*D*) Discreet cortical osteophyte formation in the epiphysis of femur illustrated by µCT (yellow dotted lines) and Kossa staining (red dotted lines). (*E*) mRNA expression of chondrogenic markers such as Sox9 transcription factor, Col II, as well as cartilage‐degrading enzymes (ADAMTS5, MMP‐3, MMP13) in paw extracts from wt (*n* = 4), placebo‐treated and anti‐TNF–treated hTNFtg mice (each *n* = 6). [Color figure can be viewed at wileyonlinelibrary.com]

We investigated TRAP and TB histological sections from small and large joints after treatment. In placebo‐treated hTNFtg mice, numerous synovial osteoclasts and massive subchondral bone erosions were present. As expected, in hTNFtg mice treated with anti‐TNF, we found a significant reduction in synovial osteoclast formation (Fig. 4 *A*, *B*) and activity (Fig. 4 *C*). Consistently, negligible signs of subchondral bone erosions were present in the treated mice (Fig. 4 *B*). Some joints showed completely intact bone architecture, whereas other joints showed areas of ongoing regenerative processes of cortical and subchondral bone tissues (Supporting Table 1). Preexisting bone erosion sites were refilled with cartilaginous or fibrocartilaginous tissue in ankle, knee, and shoulder joints of anti‐TNF–treated mice (Fig. 5 *A*). Sites of chondrogenic refillings strongly stained for collagen type II (Fig. 5 *B*). TB positively stained regeneration tissue indicating proteoglycan‐rich cartilaginous tissue was quantitatively assessed in joints from anti‐TNF–treated hTNFtg mice (Fig. 5 *C*). Of note, signs of cortical osteophyte‐like structures were partially found in knee joints as indicated by µCT. Mineralization of cortical new bone formation is shown by Kossa staining (Fig. 5 *D*). Moreover, we found an increased mRNA expression of chondrogenic markers such as Sox9 transcription factor and collagen type II in paw extracts, accompanied by a decreased expression of cartilage degrading enzymes such as ADAMT‐S5, MMP‐3, and MMP‐13 in response to anti‐TNF treatment (Fig. 5 *E*). These findings prove the partial restoration of bone erosions and initiation of regenerative processes upon therapeutic intervention with anti‐TNF.

### TNF blockade inhibits inflammation‐driven cartilage damage

Anti‐TNF treatment led to significantly lower proteoglycan loss in cartilage tissue and prevented degradation of the superficial cartilage layer compared to placebo. Moreover, preexisting lesions of calcified cartilage layers were found to be refilled with cartilaginous tissue upon TNF blockade (Supporting Fig. 4A, B).

### [^18^F]fluoride PET/CT imaging detects normalization of local bone remodeling in response to TNF blockade

**Figure 6:**
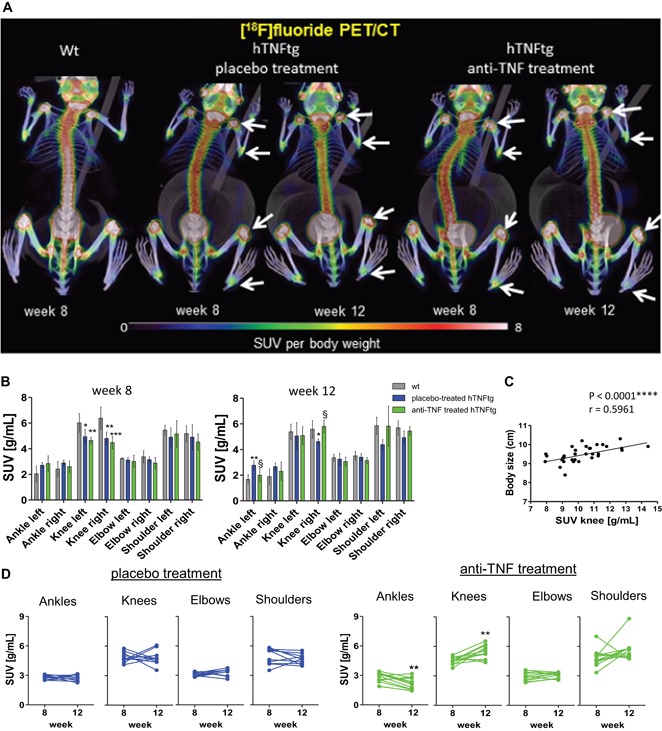
Longitudinal in vivo [^18^F]fluoride PET/CTs monitor bone remodeling. (*A*) [^18^F]fluoride accumulations before and after treatment period in the 3 groups. (*B*) Quantitative analysis of [^18^F]fluoride SUV_mean_ values before and after treatment. (*C*) Linear regression analysis for association between [^18^F]fluoride SUV_mean_ values in knees and body size. (*D*) Changes of [^18^F]fluoride SUV_mean_ values in joints on individual levels from mice after anti‐TNF or placebo treatment. Magnification: 200×, 400×. Number of animals: placebo‐treated hTNFtg, *n* = 6; anti‐TNF–treated hTNFtg, *n* = 6; wt *n* = 4. *Statistical significant compared to wt, ^§^statistical significant difference between placebo and anti‐TNF–treated group. [Color figure can be viewed at wileyonlinelibrary.com]

Based on the joints of interest and treatment, [^18^F]fluoride SUVs uptake provided inconsistent results in regard to bone remodeling. Marked [^18^F]fluoride accumulation was found in large joints such as knees, shoulders, elbows, and the spine of both hTNFtg and wt mice, primarily due to epiphyseal growth plate activity (Fig. 6 *A*; Supporting Fig. 2 and 3). Strong [^18^F]fluoride signals at growth plates overshadowed adjacent areas and prevented a separate consideration of tracer uptake at subchondral bone erosion sites. However, compared to wt animals, hTNFtg mice had a slightly lower [^18^F]fluoride uptake at growth plates related to decreased bone formation activities at sites of growth plates (Supporting Fig. 6A, B). [^18^F]fluoride SUVs in knee joints strongly correlated with the body size of animals (Pearson *r* = 0.5961, *p* < 0.0001****, Fig. 6 *C*). In the 4‐week follow‐up, [^18^F]fluoride SUVs did not markedly change in wt and placebo‐treated hTNFtg animals. Only in anti‐TNF–treated hTNFtg animals, did we observe a significant increase of [^18^F]fluoride SUVs at growth plates from knees (1.2‐fold, *p* = 0.0026) related to increased bone formation and body size (Supporting Figs. 5 and 6). When comparing the three animal groups, we observed a recurrence of [^18^F]fluoride SUVs to normality upon anti‐TNF treatment (Fig. 6 *B*, right panel) associated with increased trabecular bone mass, osteoblast numbers and osteoid formation (Supporting Fig. 6). These data suggest a major negative influence of systemic inflammation on growth plate activity and improvement upon therapy.

**Figure 7:**
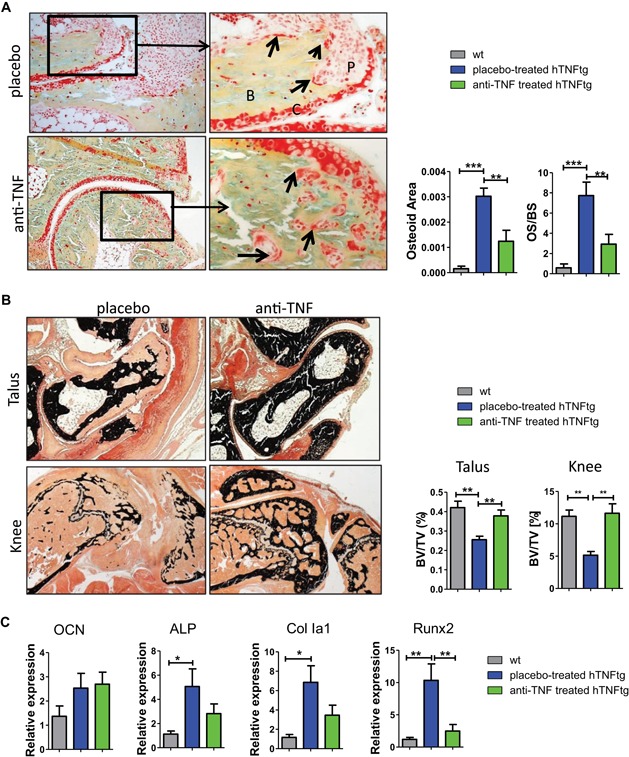
Normalization of high bone turnover upon TNF blockade in hTNFtg mice. (*A*) MOVAT‐stained sections indicate increased osteoid formation (arrows) at sites of eroded bone surfaces within ankles from placebo‐treated mice as well as sites of bone regeneration from anti‐TNF–treated hTNFtg mice. Quantitative analysis of osteoid area (mm^2^) and OS/BS in talus bone. (*B*) Representative von Kossa–stained sections indicating mineralized bone tissue (black color) of talus (upper images) and knee (lower images) from both treatment groups. Quantification of BV/TV (in %). (*C*) mRNA expression of osteogenic marker genes such as OCN, ALP, Col Ia1, and Runx2 transcription factor. Number of animals: placebo‐treated hTNFtg, *n* = 6; anti‐TNF–treated hTNFtg, *n* = 6; wt *n* = 4. Magnification: (*A*) 200×, 400×; (C) 25×, 50×. P = pannus; B = bone; C = cartilage; OS/BS = osteoid surface per bone surface; BV/TV = bone volume per tissue volume; OCN = osteocalcin; ALP = alkaline phosphatase; Col Ia1 = collagen type I. [Color figure can be viewed at wileyonlinelibrary.com]

Secondly, however, compared to age‐matched wt animals, we found a slightly increased accumulation of [^18^F]fluoride in ankle joints from hTNFtg mice (Fig. 6 *A*, *B*), which was associated with an increased presence of osteoblasts, osteoblast activity, and osteoid formation at eroded bone surfaces (Fig. 7 *A*, *C*). However, locally increased osteoid formation could not compensate for excessive bone destruction in ankles of placebo‐treated hTNFtg mice, a typical feature also found in subchondral bone erosions from RA patients (Supporting Fig. 7). Although clinical signs of arthritis progressed over time upon placebo administration, [^18^F]fluoride SUVs did not markedly change in ankle joints (Fig. 6 *B*, *D*). After 4 weeks of treatment, individual mice showed significantly reduced [^18^F]fluoride SUVs in the ankle joints (20% decrease, *p* = 0.003), indicating a normalized osteoblast activity upon TNF blockade (Fig. 6 *D*; Fig. 7 *A*, *C*). Anti‐TNF treatment led to a successful restoration of bone volume, showing complete absence of osteoclasts and reduction of osteoblast numbers and osteoid formation to levels similar to wt animals (Fig. 7 *A*, *B*).

## Discussion

In our study, we used µPET/CT imaging techniques to monitor and quantify (i) TNF‐induced chronic inflammatory polyarthritis and (ii) repair of preexisting joint damage in vivo upon therapeutic intervention. Standard evaluations of experimental arthritis models show limitations because they are generally restricted to the clinical assessment of easily accessible joints and histological analyses at predetermined endpoints without intraindividual follow‐up options. In contrast, PET/CT imaging provides a longitudinal, noninvasive, whole‐body monitoring of systemic inflammation and joint damage in the same individuals.

In our study, [^18^F]FDG showed intense accumulations in various joints of hTNFtg mice at week 8. Affected joints included shoulders, knees, ankles, and vertebrae. Based on the knowledge of partial volume effects, small inflammatory areas (< 1.2 to 1.5 mm) are not resolvable by PET, limiting the detection of inflammatory processes in small joints or lymph nodes. Further increase in [^18^F]FDG SUVs from weeks 8 to 12 indicated disease progression and increased severity of synovial inflammation, especially in knee and shoulder. Minor changes in the severity of synovitis in arthritic ankle joints have been reported.^14^, ^25^ Thus, whole‐body [^18^F]FDG imaging allows not only the identification, but even more importantly the monitoring and quantification of joint inflammation in vivo. Consistently, also in RA patients, [^18^F]FDG PET imaging studies showed a strong correlation of [^18^F]FDG uptake and metabolic activity of synovitis with disease activity.^10^, ^11^, ^26^, ^27^

Reversibility of articular inflammation upon inhibition of TNF has been documented in hTNFtg mice by clinical assessment, gait analysis, and histological analysis at the endpoint but not by in vivo imaging modalities.^14^, ^15^, ^28^ Here, we show that by performing whole‐body [^18^F]FDG imaging repeatedly, a therapeutic response can be monitored longitudinally in multiple joints of individual hTNFtg animals. Upon TNF blockade, normalization of [^18^F]FDG SUVs was achieved in various affected joints such as knee, shoulder, and ankle, showing remission of preexisting synovial inflammation in hTNFtg individuals.

Previous in vitro and in vivo studies indicated that periarticular uptake of [^18^F]FDG is predominantly mediated by activated synovial fibroblasts and macrophages.^17^ In line with these results, we have shown that the normalization of [^18^F]FDG SUVs upon TNF inhibition is associated with a marked reduction of synovial fibroblast, macrophage, and neutrophil numbers in the synovium.

Chronic joint inflammation causes dysregulated bone remodeling characterized by the promotion of bone destruction and inhibition of bone formation.^29^ Recent studies suggest repair of erosions in some RA patients treated with biologic agents such as TNF or IL‐6R inhibitors.^4^, ^30^, ^31^ Initial signs of bone repair included the presence of bony depositions or osteosclerotic changes, associated with a decrease in erosion size and improvements of radiographic scores.^4^ In experimental arthritis, bone repair has also been initiated by combination therapy of TNF inhibitors and antagonists of Wnt signaling pathway or parathyroid hormone, respectively.^32^, ^33^, ^34^ TNF blockade alone has been shown to initiate bone repair and healing processes in hTNFtg mice.^14^, ^15^ However, all these animal studies had major limitations, because bone repair had only been detected by comparative histological analysis with placebo‐treated or baseline data. In our study, longitudinal multimodal in vivo imaging enabled the direct comparison of in vivo situations before and after the treatment period in each individual animal. CT scans of anti‐TNF–treated hTNFtg mice showed significantly improved radiographic bone volumes compared to CT scans obtained before treatment. Preexisting bone erosions regenerated with varying extent up to complete restoration of bone architecture. This proves conclusively that bone repair and alleviation of preexisting bony lesions is achievable upon TNF blockade.

The mechanisms leading to this healing process are not fully understood. Repopulation of osteoblasts and the occurrence of bone repair at sites of erosions have been shown in arthritis models.^34^, ^35^, ^36^ Our in vivo data indicate that resolution of inflammatory processes is essential to allow initiation of such processes. We found bone healing processes at various sites of distinct joints: preexisting cortical and subchondral bone erosions were either completely restored (25% to 40% of joints), refilled with fibrocartilaginous tissue, or substituted by hyperproliferative chondrocytes. Only few joints showed undirected restoration by forming cartilaginous soft callus or signs of secondary osteoarthritis. Further investigations will be required to identify molecular and cellular mechanisms contributing to such aberrant regeneration processes.

In contrast to [^18^F]FDG uptake, which reflects inflammation, [^18^F]fluoride uptake reflects ion exchange within hydroxyapatite forming [^18^F]fluorapatite and thus incorporation into the skeleton at sites of active osteoblastic bone synthesis.^13^, ^37^, ^38^ Indeed, [^18^F]fluoride scans are widely used for the detection of bone metastasis and osteosarcomas.^39^ In RA patients, [^18^F]fluoride signals have been associated with affected joints, especially those with ongoing erosive changes.^40^ So far, only a single experimental arthritis study of mice immunized with G6PI demonstrated a correlation of [^18^F]fluoride accumulation with bone surface alterations as a consequence of erosive sites but also spontaneous new bone and osteophyte formation in affected paws.^18^ In contrast, in our animal model, which is an erosive arthritis model without spontaneous new bone formation, [^18^F]fluoride imaging demonstrated major limitations regarding bone remodeling changes and structural bone damage. Despite tendencies of increased [^18^F]fluoride SUVs in ankles from hTNFtg mice, which were associated with the local increase and activity of osteoblasts at sites of inflammatory bone erosions, there was no detectable difference during the investigated period compared to wt animals. On the other hand—and to our surprise—anti‐TNF therapy was associated with normalized [^18^F]fluoride SUVs in the animal's ankle joints, which might relate to a decrease in eroded bony surfaces and osteoblast activity after treatment.

Another limitation of bone‐tracers such as [^18^F]fluoride or [^99^Tc]methylene diphosphonate for our investigation is their accumulation in growth plates from rats and mice.^37^ Consistently, we observed intensive [^18^F]fluoride accumulations in large joints such as knees and shoulders in hTNFtg but also in wt mice. These [^18^F]fluoride signals reflected osteoblast activity at sites of growth plates, thereby excluding a separate consideration of adjacent epiphyseal bone erosive lesions or repair processes. Because most common arthritis models do not develop in old, skeletally mature animals, there is no alternate erosive arthritis model available to avoid this effect. Consistently, the use of an alternative method such as optical imaging with a hydroxyapatite‐targeting fluorescent marker (OsteoProbe) also showed limited sensitivity to quantify bone damage.^41^ Advances in identifying differences between physiological and pathophysiological bone remodeling are necessary for the development of novel pathology‐associated bone tracers. Overall, our study demonstrates that [^18^F]fluoride is not an appropriate tracer to quantify the extent of local inflammation‐mediated bone damage and remodeling processes in joints from this TNF‐driven arthritis model.

As a further limitation, we did not confirm the data obtained in another experimental model of arthritis, because most of the other models, such as collagen‐induced arthritis or K/BxN serum transfer arthritis are associated with spontaneous resolution and healing processes.^35^, ^42^, ^43^

In contrast to conventional radiography, which is used almost exclusively for evaluating joint damage, advances in other imaging techniques such as magnetic resonance imaging (MRI), ultrasound (US), and PET allow the simultaneous monitoring of joint inflammation and disease activity in both RA patients as well as in preclinical animal models. US has the advantage of lower costs and easy accessibility, but is limited by its acoustic window, and interobserver and intermachine reliability. In contrast, MRI may deliver valuable information about inflammation beyond the findings of other modalities (eg, bone marrow edema or contrast enhancement). Importantly, [^18^F]FDG PET is a direct readout of energy consumption, capable of detecting slight biochemical changes in “real‐time.” These changes represent active metabolic processes, which are accepted as the gold standard evidence for active inflammation. Therapeutic responses that improve inflammatory processes can therefore be readily observed as a reduction of metabolic activity and thus reduced [^18^F]FDG values. Recently, a comparative study of PET and MRI in early RA patients clearly demonstrated the superiority of PET in detecting subclinical synovitis and its potential use in predicting clinical flares.^44^ Unfortunately, a direct comparison of these techniques in murine models is limited by the scarcity of head‐to‐head studies. However, longitudinal animal studies revealed volumetric µCT imaging as a valuable quantitative tool for structural bone damage and other techniques such as Power Doppler US, contrast‐enhancement MRI, and PET as quantitative tools for monitoring synovial inflammation.^45^, ^46^, ^47^ Unfortunately, small animal MRI and PET are limited by accessibility and costs for preclinical studies. In conclusion, the primary advantage of [^18^F]FDG PET over the other imaging methods is its high specificity and value as a direct readout of energy consumption throughout the whole body. Future comparative studies utilizing different imaging techniques may one day enable researchers to provide clear recommendations on the use of such techniques in arthritis models.

To summarize, by the use of [^18^F]FDG PET/CT we could demonstrate and prove the resolution of joint inflammation to allow subsequent restoration of joint architecture upon TNF blockade in one and the same arthritic animals. Thus, multimodal [^18^F]FDG PET/CT provides a noninvasive, sensitive, objective, and quantitative in vivo tool to monitor therapeutic effects on distinct compartments such as the synovial membrane and bone in individual animals.

## Disclosures

All authors state that they have no conflicts of interest.

## Supporting information

Supporting Information.

Supporting Information.
