# Supplementary material for: Multimodal [18F]FDG PET/CT Is a Direct Readout for Inflammatory Bone Repair: A Longitudinal Study in TNFα Transgenic Mice
Source: J Bone Miner Res. 2019 Jul 30;34(9):1632–45. doi: 10.1002/jbmr.3748 (PMC6852546; doi:10.1002/jbmr.3748)

**SUPPORTING INFORMATION**

**
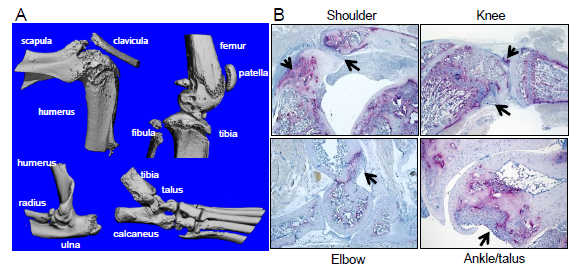
**

**Supporting Figure 1: Disease severity of hTNFtg animals before treatment at week 8 of age.** (A) Representative µCT images demonstrated presence of bone erosions in small and large joints from hTNFtg mice. (B) Histological analysis from TRAP-stained sections showed synovial inflammation, invasive pannus formation, synovial osteoclasts and bone erosions (arrow) in various small and large joints such as shoulder, knee, elbow and ankle. Magnification is 50x and 100x.


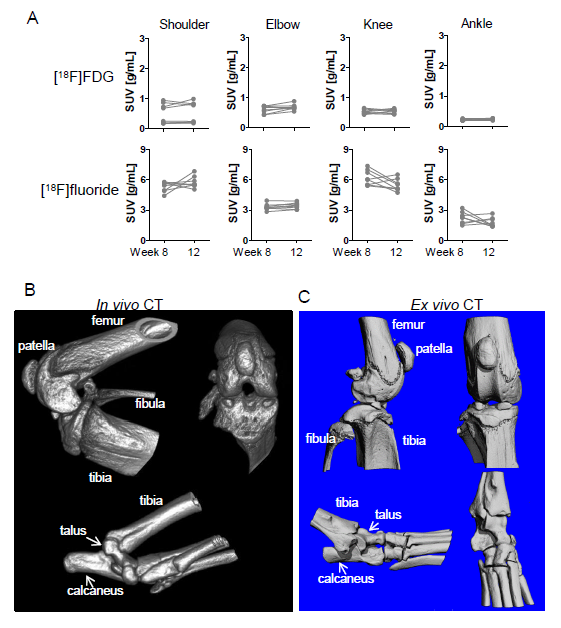


**Supporting Figure 2: Radiotracer uptake and CT images of joints from wt mice**. Quantitative analysis of [^18^F]FDG and [^18^F]fluoride SUV_mean_ values in joints from wt individuals at week 8 and 12 of age. (B) Representative *in vivo* CT images from knee (upper images) and hind paw (ankle). (C) *Ex vivo* CT images from knee (upper images) and hind paw (ankle, lower images).


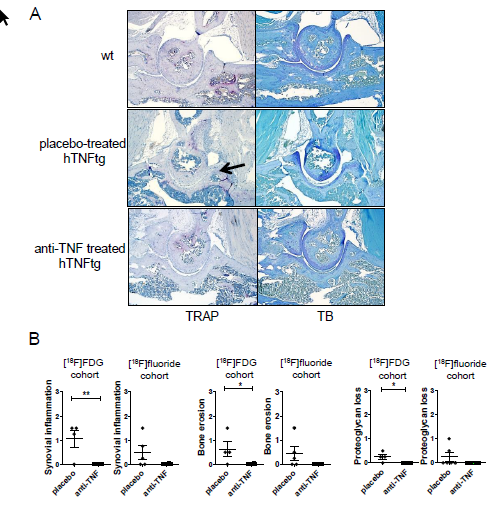


**Supporting Figure 3:** **Histopathological signs of arthritis in elbow joints from anti-TNF ab treated hTNFtg, placebo-treated hTNFtg and wt mice at week 12.** (A) Representative images from TRAP stained sections (left column) indicated low presence of synovial osteoclasts (purple-colored cells), rare bone erosions and mild synovial inflammation (arrow). TB stained sections (right column) illustrated mild signs of cartilage damage in placebo-treated hTNFtg mice. Anti-TNF treated mice showed no signs of inflammatory, erosive arthritis in elbow joints. Original magnification is 100x. (B) Semi-quantitative analysis of synovial inflammation, bone erosion, proteoglycan loss of articular cartilage.


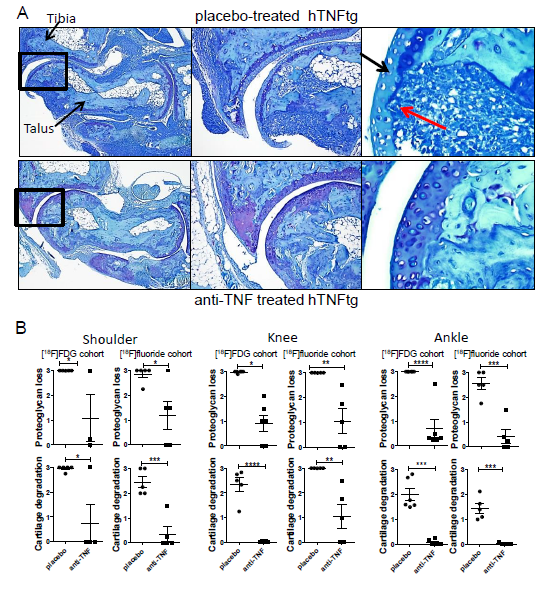


**Supporting Figure 4:** **Anti-TNF treatment blocks progressive inflammatory cartilage damage in hTNFtg mice**. (A) Representative images from TB stained sections showed inflammation-mediated cartilage damage indicated by proteoglycan loss (destaining of TB) of superficial cartilage layer (black arrow) and degradation of underlying calcified cartilage (red arrow) in placebo-treated hTNFtg (upper row). Upon TNF blockade increased proteoglycan loss as well as refilled calcified cartilage tissue was observed. (B) Semi-quantitative analysis of proteoglycan loss and cartilage degradation in various joints such as shoulder, knee and ankle joints after placebo or anti-TNF treatment. Magnification is 50x (left), 100x (middle) and 200x (right).

**
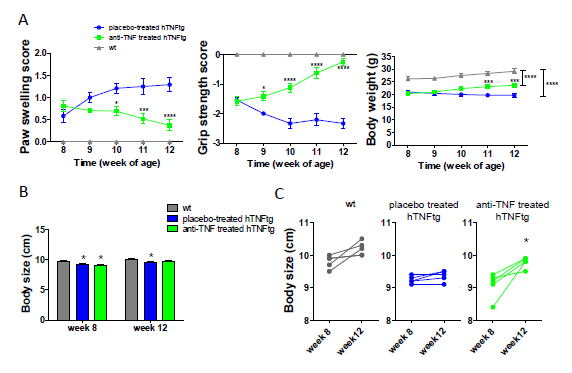
**

**Supporting Figure 5: Clinical course and body size of [^18^F]fluoride** **cohort including anti-TNF treated hTNFtg and placebo treated hTNFtg mice and wt littermates.** (A) Clinical course of arthritis signs including paw swelling and loss of grip strength as well as body weight during treatment period from week 8 to week 12. (B) Comparison of body sizes (in cm, body length) between wt, anti-TNF treated hTNFtg and placebo treated hTNFtg mice at week 8 and at week 12. (C) Body size changes from week 8 to week 12 in the individual animal groups.* statistical significance compared to wt.


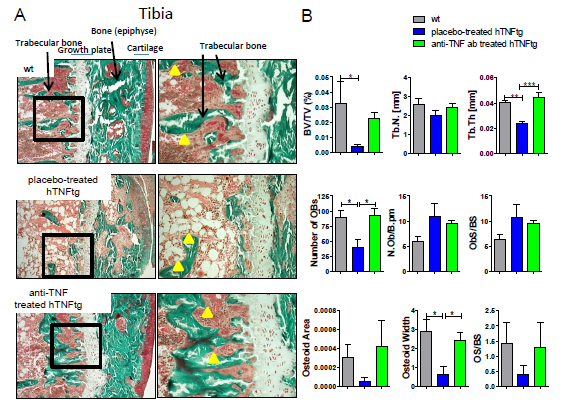


**Supporting Figure 6**: **Systemic osteoblast activity.** (A) Goldner stained sections indicated presence of osteoblasts (yellow triangles) and osteoid formation at growth plates from tibiae of wt, placebo and anti-TNF treated hTNFtg mice. (B) Histomorphometric analysis of following bone parameters was evaluated in trabecular bone: bone volume per tissue volume (BV/TV, in %), trabecular thickness (Tb.Th.), trabecular number (Tb.N.), number of osteoblasts, number of osteoblasts per bone perimeter (N.Ob/B.pm), osteoblast surface per bone surface (ObS/BS), osteoid area, osteoid width and osteoid surface per bone surface (OS/BS). Original magnification 200x and 400x.


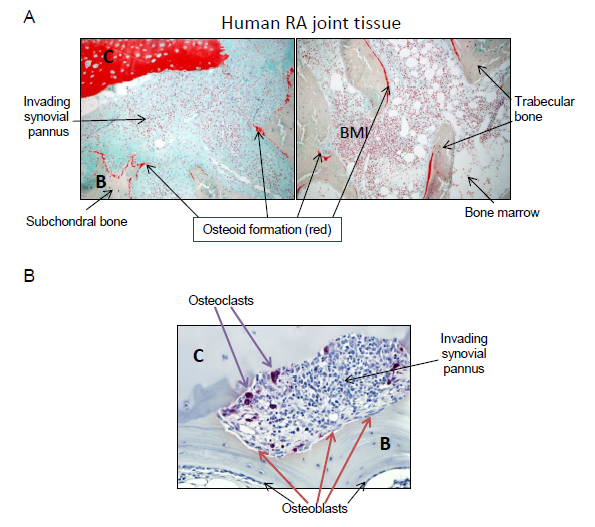


**Supporting Figure 7. Presence and activity of osteoblasts within subchondral bone erosion from RA patients.** (A) Representative image of subchondral bone erosion indicating invading synovial pannus tissue, bone erosion and calcified cartilage destruction. MOVAT staining illustrated unique osteoid formation (red accumulations, arrows) at eroded subchondral bone surfaces (left image) as well as at endosteal areas next to bone marrow infiltrates (red accumulations, arrows, right image). (B) TRAP staining indicates bone resorbing osteoclasts at sites of calcified cartilage and bone tissue invaded by inflammatory synovial pannus. Osteoblasts are localized at eroded bone surfaces within subchondral bone erosions (red arrows) or at endosteal bone (black arrows). C…cartilage, B…bone, BMI…bone marrow infiltrate. Original magnification is 100x (A) and 200x (B).

**Supporting Table:** Table summarizes the number of joints (in %) with intact bone architecture or ongoing bone regeneration processes in anti-TNF treated hTNFtg mice.


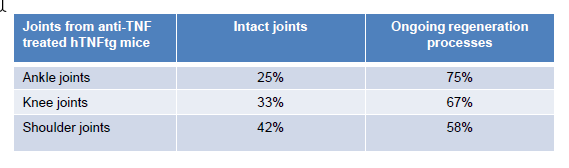

Supplement: Supplementary file 1 — Supporting Information. [file JBMR-34-1632-s001.docx]
