# Supplementary material for: Multimodal [18F]FDG PET/CT Is a Direct Readout for Inflammatory Bone Repair: A Longitudinal Study in TNFα Transgenic Mice
Source: J Bone Miner Res. 2019 Jul 30;34(9):1632–45. doi: 10.1002/jbmr.3748 (PMC6852546; doi:10.1002/jbmr.3748)
Supplement: Supplementary file 2 — Supporting Information. [file JBMR-34-1632-s002.docx]

**Supporting Methods:**

Total RNA from front paws was extracted by mechanical homogenization (TissueLyser,) using Trizol reagent (Invitrogen, Carlsbad, CA) according to the manufacturer’s protocol. After cDNA synthesis (Omniscript Reverse transcription kit, Qiagen), real-time PCR was performed with SYBR Green I Master and a Lightcycler 480 (Roche Molecular Systems, Inc). Relative expression was calculated using the 2−ΔΔCt method by normalizing with GADH housekeeping gene expression.

**Primer sequences for Real-time PCR:**

Forward Reverse

GAPDH 5’-TGGCATTGTGGAAGGGCTCATGAC-3’ 5’-ATGCCAGTGAGCTTGCCGTTCAGC-3’

ADAMTS5 5’-GGAGCGAGGCCATTTACAAC-3’ 5’-CGTAGACAAGGTAGCCCACTT T -3’

ALKALINE PHOSPHATASE 5’-cacgcgatgcaacaccactcagg-3’ 5’-gcatgtccccgggctcaaaga-3’

CATHEPSIN K 5’-GGAAGAAGACTCACCAGAAGC-3’ 5’-GTCATATAGCCGCCTCCACAG-3’

COLLAGEN TYPE Ia1 5’-CTG ACT GGA AGA GCG GAG AG-3’ 5’-GCA CAG ACG GCT GAG TAG G-3’

COLLAGEN TYPE II 5’-GCG AGA GGG GAC TGA AGG GAC ACC-3’ 5’-CGG GGC TGC GGA TGC TCT CAA T-3’

OSTEOCALCIN 5’-accctggctgcgctctgtctct-3’ 5’-gatgcgtttgtaggcggtcttca-3’

MMP-3 5’-CGATGATGAACGATGGACAG-3’ 5’-AGCCTTGGCTGAGTGGTAGA-3’

MMP-9 5’-CCTGTGTGTTCCCGTTCATCT-3’ 5’-CGCTGGAATGATCTAAGCCCA-3’

MMP-13 5’-AAAGATTATCCCCGCCTCAT-3’ 5’-TGGGCCCAT TGAAAAAGTAG-3’

RUNX2 5’-cggagcggacgaggcaagagtttc-3’ 5’-agacagcggcgtggtggagtggat-3’

SOX9 5’-TCTGGAGGCTGCTGAACG A-3’ 5’-TCCGTTCTTCACCGACTTCCT-3’

TRAP 5’-ACAGCCCCCACTCCCACCCT-3 5’-TCAGGGTCTGGGTCTCCTTGG-3’
